# Differential Proteomic Expression of Equine Cardiac and Lamellar Tissue During Insulin-Induced Laminitis

**DOI:** 10.3389/fvets.2020.00308

**Published:** 2020-06-12

**Authors:** Allison Campolo, Matthew W. Frantz, Melody A. de Laat, Steven D. Hartson, Martin O. Furr, Véronique A. Lacombe

**Affiliations:** ^1^Department of Biochemistry and Molecular Biology, Center for Veterinary Health Sciences, Oklahoma State University, Stillwater, OK, United States; ^2^Biosciences, Queensland University of Technology, Brisbane, QLD, Australia

**Keywords:** laminitis, endocrinopathic, proteomic, equine metabolic syndrome, heart, lamellar

## Abstract

Endocrinopathic laminitis is pathologically similar to the multi-organ dysfunction and peripheral neuropathy found in human patients with metabolic syndrome. Similarly, endocrinopathic laminitis has been shown to partially result from vascular dysfunction. However, despite extensive research, the pathogenesis of this disease is not well elucidated and laminitis remains without an effective treatment. Here, we sought to identify novel proteins and pathways underlying the development of equine endocrinopathic laminitis. Healthy Standardbred horses (*n* = 4/group) were either given an electrolyte infusion, or a 48-h euglycemic-hyperinsulinemic clamp. Cardiac and lamellar tissues were analyzed by mass spectrometry (FDR = 0.05). All hyperinsulinemic horses developed laminitis despite being previously healthy. We identified 514 and 709 unique proteins in the cardiac and lamellar proteomes, respectively. In the lamellar tissue, we identified 14 proteins for which their abundance was significantly increased and 13 proteins which were significantly decreased in the hyperinsulinemic group as compared to controls. These results were confirmed via real-time reverse-transcriptase PCR. A STRING analysis of protein-protein interactions revealed that these increased proteins were primarily involved in coagulation and complement cascades, platelet activity, and ribosomal function, while decreased proteins were involved in focal adhesions, spliceosomes, and cell-cell matrices. Novel significant differentially expressed proteins associated with hyperinsulinemia-induced laminitis include talin−1, vinculin, cadherin-13, fibrinogen, alpha-2-macroglobulin, and heat shock protein 90. In contrast, no proteins were found to be significantly differentially expressed in the heart of hyperinsulinemic horses compared to controls. Together, these data indicate that while hyperinsulinemia induced, in part, microvascular damage, complement activation, and ribosomal dysfunction in the lamellae, a similar effect was not seen in the heart. In brief, this proteomic investigation of a unique equine model of hyperinsulinemia identified novel proteins and signaling pathways, which may lead to the discovery of molecular biomarkers and/or therapeutic targets for endocrinopathic laminitis.

## Introduction

Alongside their human counterparts, the incidence of metabolic syndrome and obesity in horses has been steadily rising. Recent studies find that 32% of surveyed horses in the United States are overweight, and 19% are clinically obese (1). As endocrinopathic laminitis has been shown to be linked with equine metabolic syndrome and obesity, the frequency of laminitis is estimated to be as high as 34% in populations of horses in Western countries (2).

Similar to peripheral neuropathy in human patients with metabolic syndrome, endocrinopathic laminitis has been linked to vascular dysfunction (3, 4). In addition, metabolic diseases in other mammals have been found to increase the prevalence of cardiac diseases (e.g., diastolic dysfunction, heart failure, stroke, and arrhythmia), along with alterations in glucose transport and its downstream insulin signaling pathway in cardiac muscle (5, 6). However, whether similar cardiac complications could occur during equine metabolic syndrome is not well unknown.

While some progress has been made in a traditional, protein-by-protein approach in the endeavor of understanding the metabolic and inflammatory pathways (2, 7–9), and the stability of lamellar integrins (10, 11), the pathophysiological mechanisms underlying endocrinopathic laminitis are not well understood. Importantly, as there is still no known definitive or singular cause of endocrinopathic laminitis, nor is a known cure for laminitis after its onset (12). This is germane to the fact that there are few proteomic studies of laminitis. Previously, the cytoskeleteal proteins of the lamellar proteome were found to be predominantly characterized by novel keratins (13). Separately, Steelman et al. demonstrated a significant alteration in the plasma proteome of laminitic horses (14). Clotting factors such as fibrinogen and factor X were increased in the plasma of laminitic horses, while there was an increase of alpha-2-macroglobulin (14). However, animals used in this study were clinical cases of chronic laminitis, with no particular type of laminitis reported (e.g., endocrinopathic, supporting limb, etc.), and some were being treated with phenylbutazone (14). To our knowledge, no proteomic investigations of lamellar and cardiac tissue have been performed in a model of insulin-induced laminitis. Here, using a modern bottom-up proteomics approach, we identified novel effectors and potential biomarkers during insulin-induced laminitis.

## Materials and Methods

### Animal Model

**Figure 1:**
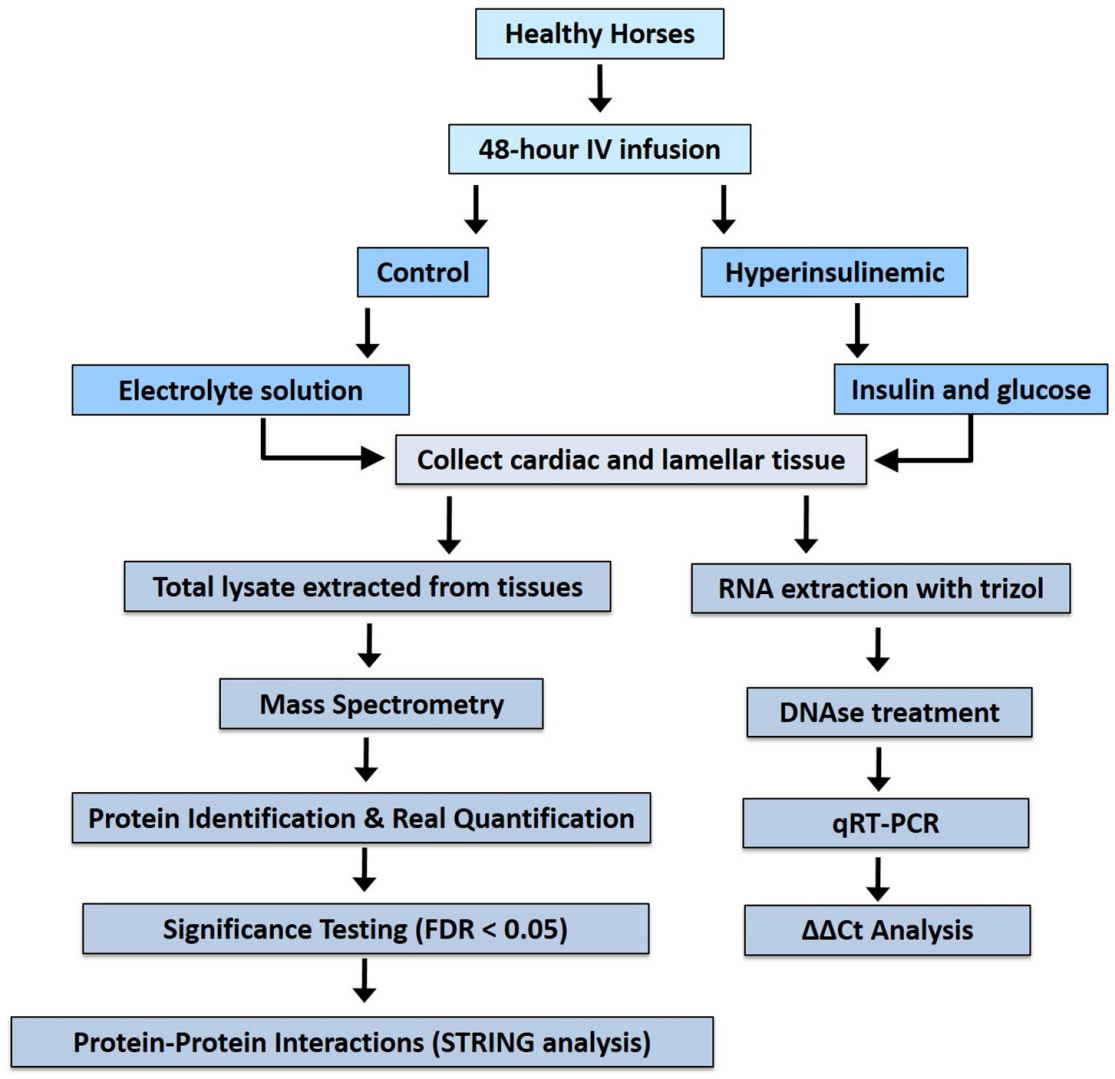
Work flow for proteomic analysis of tissues from hyperinsulinemic horses and real-time reverse-transcriptase analysis.

Archived cardiac and lamellar tissue were collected from healthy Standardbred horses (mean age of 5.4 ± 1.95 years, body condition scores 2.5–4.5/9, consisting of 7 geldings and 1 filly) (15). After physical examination, animals were found to possess normal hematology, biochemistry, and urinalysis results (15). Following 24 h of acclimatization to temperature-controlled stables, horses (*n* = 4/group) received either a prolonged insulin infusion (6 mIU/kg/min, Humulin R insulin) for 46 ± 2.3 h to induce marked hyperinsulinemia (mean serum insulin, 1,036 ± 129 μIU/mL) or a balanced electrolyte solution infused at the same rate (mean serum insulin, 10 ± 0.9 μIU/mL) as previously described (15). Despite being previously healthy, all hyperinsulinemic horses developed laminitis, whereas the control group did not. Cardiac and lamellar samples were collected and total lysates (for mass spectrometry) or RNA (for real-time reverse transcriptase PCR) were extracted (Figure 1) as previously described (15). These procedures were approved by the Animal Ethics Committee of the University of Queensland (SVS/013/08/RIRDC).

### Proteomic Analysis

Tissues were homogenized in RIPA buffer containing protease inhibitor cocktail, and protein contents were quantified by bicinchoninic assay, as previously described (6). Aliquots (25 micrograms) of each lysate were precipitated with TCA/acetone, redissolved and reduced for 1 h in 8 M urea, 5 mM TECP, 100 mM Tris pH 8.5, and then alkylated for 20 min by adding 10 mM IAA. Reactions were then diluted to 2 M urea, trypsin was added to 4 μg/ml, and digestions were incubated overnight at 37°C. After digestion, peptides were desalted by solid phase extraction on monolithic C18 pipet tips, and lyophilized to dryness.

For LC-MS/MS, one microgram of peptides was injected onto a 0.15 × 50 mm vented trap column, followed by separation on a 0.075 × 400 mm Picofrit column/emitter (New Objective). Both columns were packed with 3-micron Magic AQ C18 particles (Bruker). Unique peptides were separated at room temperature using a 5–40% ACN/0.1% formic acid gradient performed over 100 min at a flow rate of 250 nL/min, eluting directly into a New Objective PV-550 nanoelectrospray ion source. Peptides were analyzed using an LTQ OrbitrapXL mass spectrometer (Thermo) via a Top Six data-dependent acquisition method as previously described (16).

Proteins were identified and quantified by using MaxQuant (17) to search the raw instrument files against a database of 22,718 reference equine protein sequences downloaded from UniProt on January 24, 2020. Searches used the fixed modification carbamidomethyl (C) and the following variable modifications: oxidized (M), acetyl (protein N-terminus) and glutamine cyclization to glutamate). Searches were performed using default MaxQuant settings, with the addition of label-free quantitation via the LFQ algorithm and match between runs to transfer MS/MS identifications between LC-MS/MS data files.

Statistical analyses were performed using the Perseus software package (Max Planck Institute of Biochemistry) (18) to analyze log (2)-transformed MaxQuant LFQ protein intensities after filtering potential contaminants. Samples were identified as being significantly differentially expressed via Student's *t*-test using the Benjamini-Hochberg multiple tests correction, with a false discovery rate of 0.05 (19). Protein-protein interactions and KEGG (Kyoto Encyclopedia of Genes and Genomes) pathways were identified via STRING 10.5 (Search Tool for the Retrieval of Interacting Genes/Proteins).

### Quantitative Real-Time Polymerase Chain Reaction Analyses (qRT-PCR)

Frozen lamellar samples (50–80 mg) were pulverized using Trizol reagent (Invitrogen, CA, USA) according to the manufacturer's instructions and RNA quantified via absorbance (A_260_) using Gen5 software with Biotek synergy HT hardware on a take3 plate (BioTek, VT, USA). Purity was assessed via the A_260_/A_280_ ratio. DNAse-treated RNA (Ambion AM1906M) was then converted to complementary DNA using the High Capacity cDNA Reverse Transcription Kit and random primers (Applied Biosystems, CA, USA) according to manufacturer's recommendations and stored at −80°C until analysis. The qRT-PCR assays for relative quantification of β-actin, vinculin, talin-1, cadherin-13, heat shock protein 90, fibrinogen beta, and alpha-2-macroglobulin were performed using SYBR Green Real Time PCR Master Mix containing AmpliTaq Gold DNA Polymerase, to minimize nonspecific product formation, and deoxyribose nucleotide triphosphates with deoxyribose uridine triphosphate, to reduce carryover contamination (Applied Biosystems). At least two pairs of primers were designed for each target gene, with a target melting point between 60 and 62°C and spanning an exon-exon junction. Primer sequences were selected using the National Center for Biotechnology Information Basic Local Alignment Search Tool (NCBI BLAST) and custom synthesized by Invitrogen (GenBank accession numbers: β-actin: XM_023655002.1; Vinculin: XM_014733025.2; Cadherin-13: XM_02363868.1; Talin-1: XM_014735894.2, Heat Shock Protein 90: NM_001081938.1; Fibrinogen: XM_003364535.4; Alpha-2-Macroglobulin: XM_001499123.5, Table 1). Each PCR reaction (20 μL) contained 2x reaction buffer (SYBR Green I dye, Amplitaq Gold DNA Polymerase, deoxyribose nucleotide triphosphates with deoxyribose uridine triphosphate, passive reference, and optimized buffer components), forward and reverse primers (0.5 mM), 0.5 μg of complementary DNA, and DNase-RNase-free water. Primer concentrations and PCR conditions were determined during initial optimization runs. Samples were run in duplicate in a 96-well MicroAmp optical plate (Invitrogen). qRT-PCR was performed in an ABI 7500 Fast instrument (Applied Biosystems) with the following cycling conditions: 10 min at 95°C, followed by 40 cycles at 95°C for 15 s and 60°C for 1 min. No-template, negative controls were included for each gene. A melting curve was generated to ensure product purity and the absence of primer dimers. Primers were further confirmed via amplification. The messenger RNA (mRNA) expression of target genes was normalized to β-actin, and relative gene expression was quantified using the ΔΔCT method (20).

**Table 1:** Primer sequences.

**Gene Name**	**Sequence (5′-3′)**	**Product Size**
Beta Actin Forward	ATG ATG ATA TCG CCG CGC TC	131 base pairs
Beta Actin Reverse	CCA CCA TCA CGC CCT GG	
Vinculin Forward	GTC CAG CAA GCC GGG TAA C	140 base pairs
Vinculin Reverse	CCG GCT GAT TGG ATG GCAT T	
Cadherin-13 Forward	GCC GCG TGC ATG AAT GAA A	142 base pairs
Cadherin-13 Reverse	TGT TAG CAT CAG CAC CTG GG	
Talin-1 Forward	ATC GCA GAT ATG CTT CGG GC	111 base pairs
Talin-1 Reverse	GGC TTC TGC AGG GTC AGT AG	
Heat Shock Protein 90 Forward	CTT GAG TCA CCT CGC GCA	100 base pairs
Heat Shock Protein 90 Reverse	CCT CAG GCA TCT TAA CGG GC	
Fibrinogen Beta Forward	ATT CAG AAC CGC CAG GAT GG	108 base pairs
Fibrinogen Beta Reverse	ATA CTC ACC TGG TAG GCC ACA	
Alpha-2-Macroglobulin Forward	ACT CCA GAG GCC AGA TCC AA	105 base pairs
Alpha-2-Macroglobulin Reverse	TGT GAG CCA GGT ATT GCC CT	

### Statistical Analyses

Differences in expression were validated by log2 transformation of LFQ values of samples from control and hyperinsulinemic samples using Students *T*-test, wherein *p*-value thresholds were validated by a Benjamini-Hochberg test at an FDR threshold of 0.05. qRT-PCR data were generated using relative quantification via the delta delta Ct method, which was analyzed using 2-tailed Student *t*-tests. All data were expressed as mean ± standard error of the mean or median (interquartile range), and significance was accepted at *P* < 0.05.

## Results

**Figure 2:**
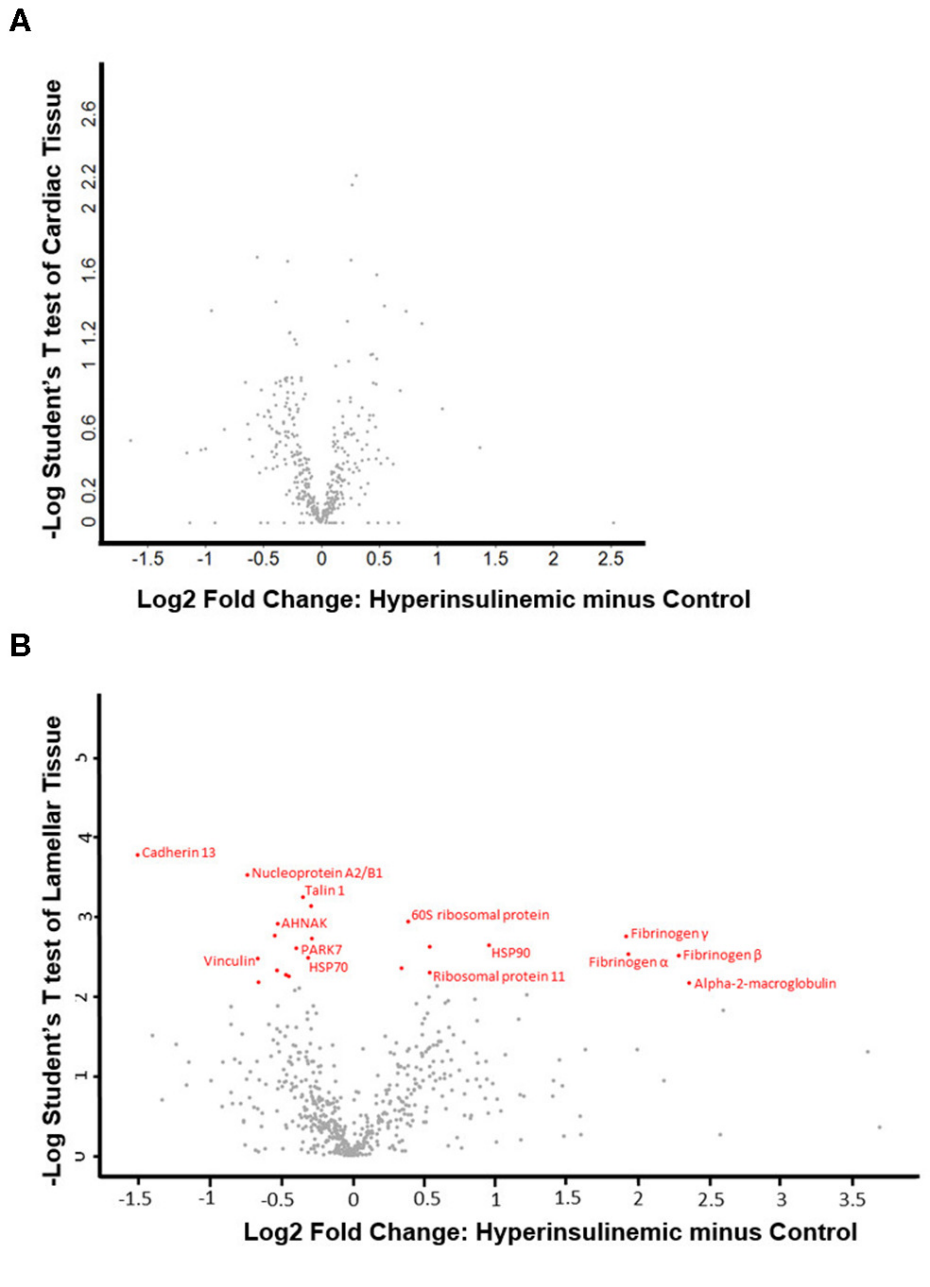
Volcano plots of **(A)** cardiac tissue and **(B)** lamellar tissue of control and hyperinsulinemic horses. Significantly differentially expressed proteins (using an FDR of 0.05) are identified in red, with the corresponding protein identification (*n* = 4/group).

**Figure 3:**
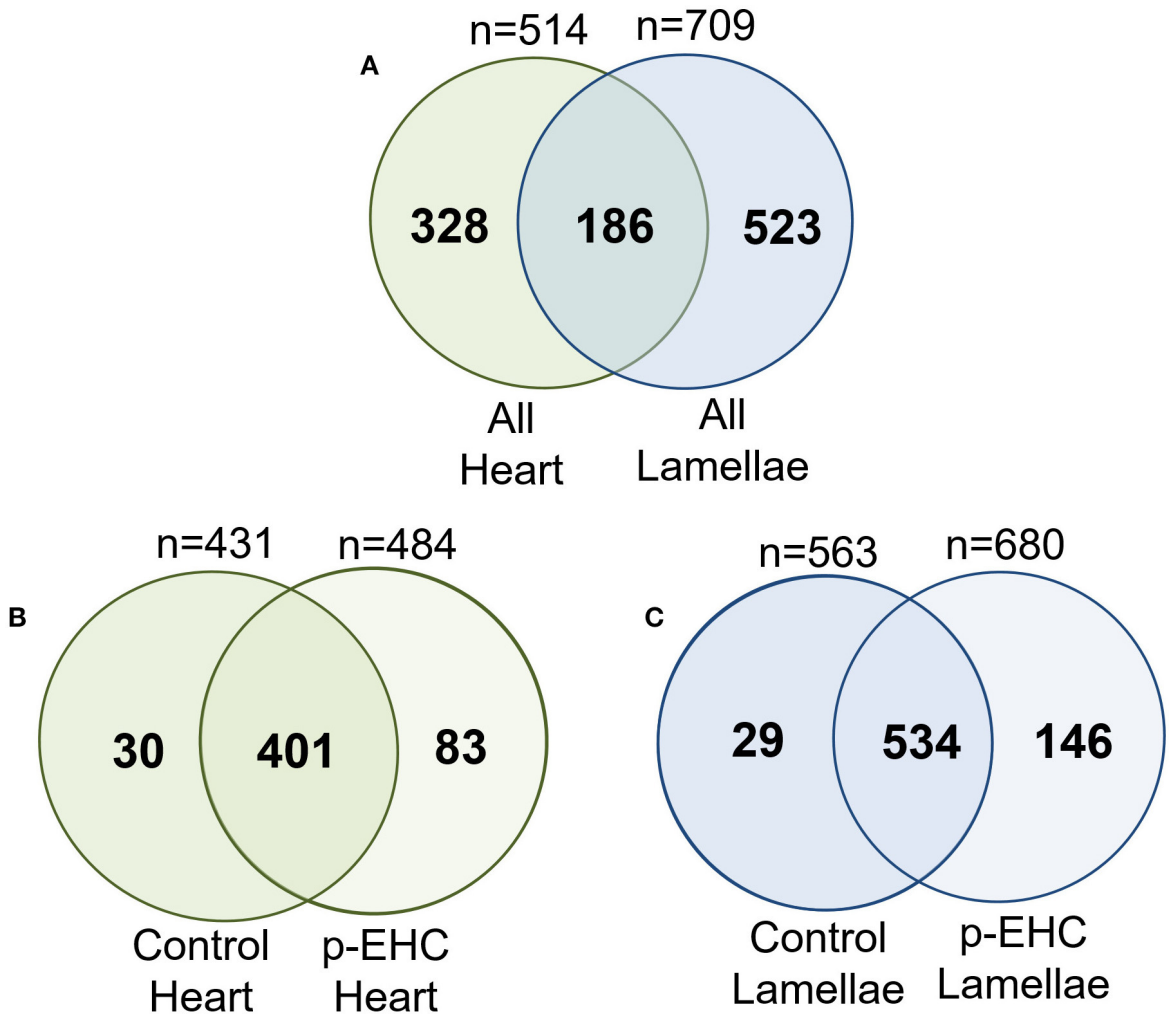
Venn analysis of all identified proteins from the cardiac and lamellar tissues of control and hyperinsulinemic (p-EHC) horses identified overlap in protein groups between **(A)** all heart and all lamellae samples, **(B)** control heart and hyperinsulinemic heart samples, and **(C)** control lamellae and hyperinsulinemic lamellae samples. *n* = 4/group.

**Figure 4:**
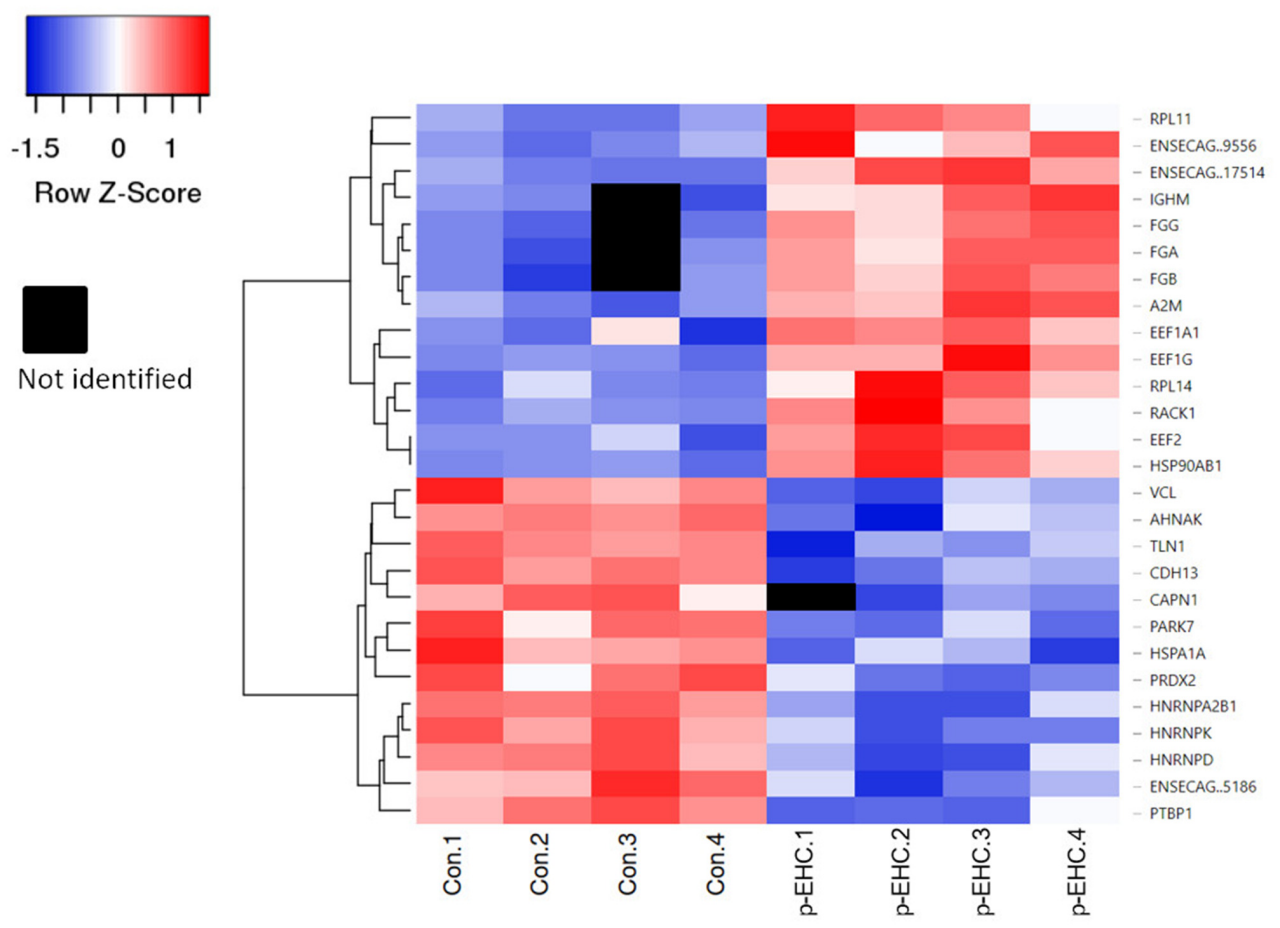
Heat map of significantly differentially expressed proteins in control (Con 1-4) and hyperinsulinemic (p-EHC 1-4) animals. Clustering indicates grouping of proteins by similar expression patterns relative to the mean distance between all objects of the clusters. Red indicates a higher expression, blue indicates a lower expression, while white indicates the least difference between groups and black indicates a lack of data for a specific protein.

The proteomic data was evenly distributed based on histogram visualization after analysis of LFQ intensity. We detected 514 and 709 proteins in the cardiac and lamellar proteomes, respectively. We did not identify any proteins which were significantly differentially expressed between the control and treatment groups in cardiac tissue, but we did identify 27 proteins which were significantly differentially expressed between groups in lamellar tissue (as indicated in red, Figure 2). From this proteomic analysis, we identified both unique and shared proteins in cardiac and lamellar samples of hyperinsulinemic and control horses (Figure 3). In the lamellar samples, detected 13 proteins for which their abundance were significantly decreased in the treatment group (Table 2), and 14 proteins which were significantly increased in the treatment group (Table 3) (FDR < 0.05). The majority of the significantly differentially expressed proteins were found to be either increased or decreased among the lamellar samples and the differential expression of these proteins were visualized via heat map (Figure 4).

**Table 2:** Proteins significantly decreased in lamellar tissue of hyperinsulinemic horses.

**Accession number**	**Uniprot protein name (Gene name)**	***P*-value**	**log2 ratio (Hyperinsulinemic-Control)**
F6UGL6	Polypyrimidine tract binding protein 1 (**PTBP1**)	0.002	0.820
F6Q4Q1	Heterogeneous nuclear ribonucleoprotein K (**HNRNPK**)	<0.001	0.819
F7DW69	Heat shock 70 kDa protein 1A (**HSPA1A**)	0.003	0.805
F6QIZ4	Talin 1 (**TLN1**)	0.001	0.786
F6WPB4	Parkinsonism associated deglycase (**PARK7**)	0.002	0.762
F6S6S0	Uncharacterized protein (**ENSECAG00000005186**)	0.005	0.725
F7AJD4	AHNAK nucleoprotein (**AHNAK**)	0.003	0.696
F6SU04	Calpain 1 (**CAPN1**)	0.004	0.695
F7BFT1	Peroxiredoxin 2 (**PRDX2**)	0.004	0.694
F6WHS3	Heterogeneous nuclear ribonucleoprotein D (**HNRNPD**)	0.002	0.687
F6ZSZ5	Vinculin (**VCL**)	0.002	0.632
F6VYB1	Heterogeneous nuclear protein A2/B1 (**HNRNPA2B1**)	<0.001	0.601
F6VN96	Cadherin 13 (**CDH13**)	<0.001	0.352

*Proteins were identified via LFQ intensity and analyzed through Perseus (P-value was calculated with a false discovery rate of 0.05 to minimize type II errors). Protein and gene names were derived from Uniprot KB database. log2 ratio is represented as treatment minus control, and calculated as log2(hyperinsulinemic/control). n = 4/group*.

**Table 3:** Proteins significantly increased in lamellar tissue of hyperinsulinemic horses.

**Accession number**	**Uniprot protein name (Gene name)**	***P*-value**	**log2 ratio (Hyperinsulinemic-Control)**
H9GZN9	Immunoglobulin heavy constant mu (**IGHM**)	0.006	12.232
F6RI47	Alpha-2-macroglobulin (**A2M**)	0.001	6.071
F6PH38	Fibrinogen beta chain (**FGB**)	0.002	4.878
H9GZU9	Uncharacterized protein (**ENSECAG00000009556**)	0.006	3.997
F6RUZ6	Fibrinogen alpha chain (**FGA**)	0.002	3.819
F6W2Y1	Fibrinogen gamma chain (**FGG**)	<0.001	3.784
Q9GKX8	Heat shock protein HSP 90-beta (**HSP90AB1**)	<0.001	1.940
F7AWX3	WD_REPEATS_REGION domain-containing protein (**RACK1**)	0.005	1.590
F6UUS3	Eukaryotic translation elongation (**EEF2**)	0.005	1.507
F6UB53	Ribosomal protein 11 (**RPL11**)	0.003	1.455
A2Q127	Elongation factor 1-gamma (**EEF1G**)	<0.001	1.454
F6STU8	Ribosomal protein 14 (**RPL14**)	0.006	1.452
F6QF58	60S ribosomal protein L6 (**ENSECAG00000017514**)	<0.001	1.312
F6UME7	Elongation factor 1-alpha (**EEF1A1**)	0.006	1.270

*Proteins were identified via LFQ intensity and analyzed through Perseus (P-value was calculated with a false discovery rate of 0.05 to minimize type II errors). Protein and gene names were derived from Uniprot KB database. log2 ratio is represented as treatment minus control, and calculated as log2(hyperinsulinemic/control). n = 4/group*.

**Figure 5:**
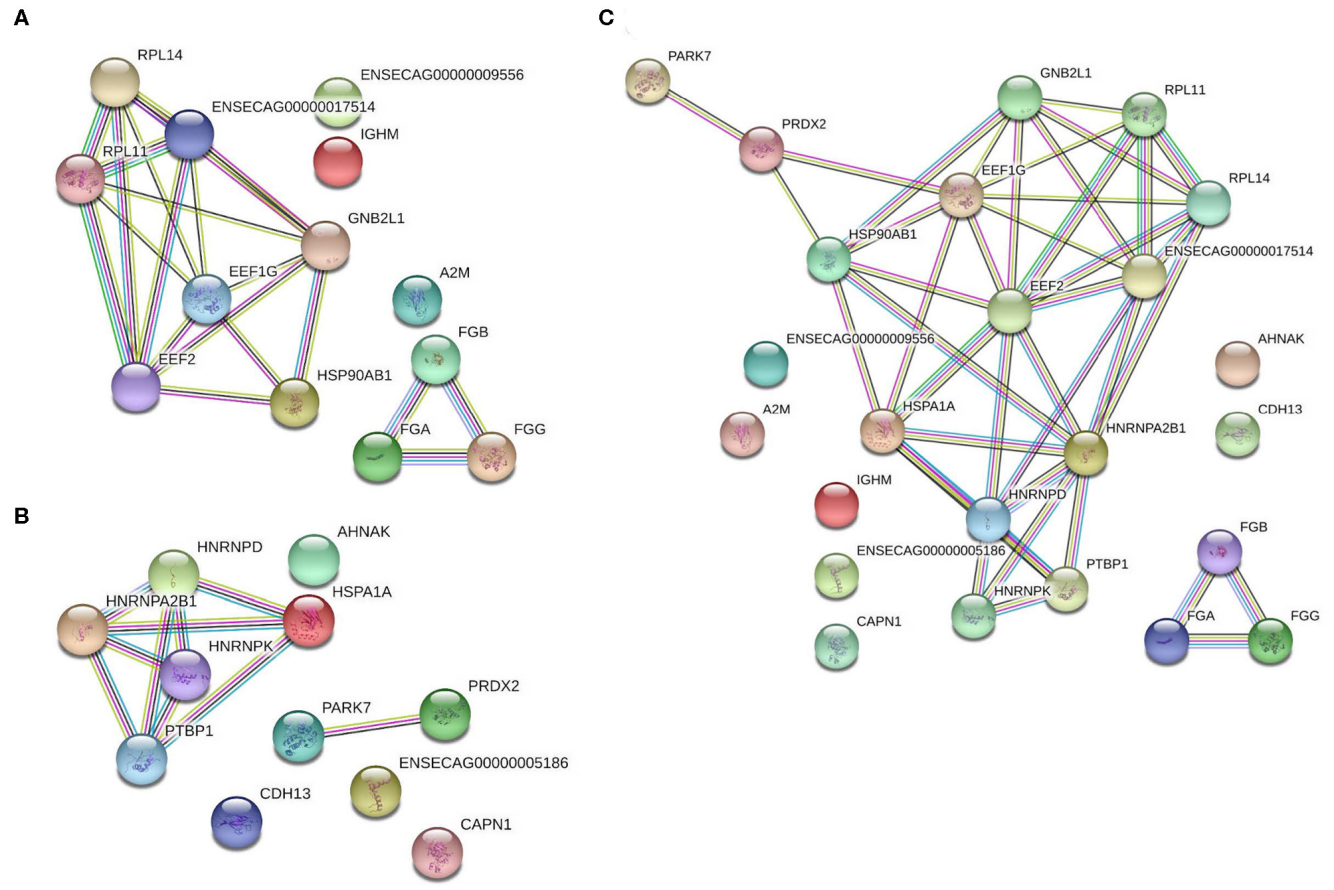
Protein-protein interaction analysis of significantly differentially expressed proteins in the lamellar tissue of hyperinsulinemic horses as compared to controls. **(A)** Proteins significantly increased in hyperinsulinemic horses, **(B)** proteins significantly decreased in hyperinsulinemic horses, and **(C)** all significantly differentially expressed proteins, together. Nodes (circles) represent proteins, while edges (lines between nodes) represent protein-protein interactions. Color code for edge interactions: known interactions from: teal, curated databases; magenta, experimentally determined; predicted interactions: green, gene neighborhood; red, gene fusions; blue, gene co-occurrence; other: yellow, textmining; black, co-expression; purple, protein homology. For protein identification lists, reference accession numbers of Tables 2, 3.

To determine the functions of these differentially expressed proteins, they were analyzed via the Search Tool for the Retrieval of Interacting Genes/Proteins (STRING) database to determine the known and predicted protein-protein interactions (Figure 5). Of the significantly differentially expressed lamellar proteins, those increased in the treatment group were primarily involved in coagulation and complement cascades, platelet activation, and ribosomal function. The proteins which were significantly decreased in the treatment group were predominately involved in focal adhesions and spliceosomes (Table 4).

**Table 4:** Significantly differentially expressed pathways in lamellar tissue of hyperinsulinemic horses.

**Pathway ID**	**Pathway description**	**Count in gene set**	**False discovery rate**
**Molecular Function (GO)**			
GO:0003723	RNA binding	2	0.05
**Cellular Component (GO)**			
GO:0070062	Extracellular exosome	2	0.038
**KEGG Pathways**			
04610	Complement and coagulation cascades	4	<0.001
03010	Ribosome	3	0.049
04611	Platelet activation	3	0.049
05162	Measles	3	0.049
**PFAM Protein Domains**			
PF08702	Fibrinogen alpha/beta chain family	3	<0.0001
PF00147	Fibrinogen beta and gamma chains, C-terminal globular domain	3	0.008

*Proteins were identified via LFQ intensity and analyzed through Perseus (FDR-0.05) to determine which proteins were significantly differentially expressed in lamellar tissue of hyperinsulinemic horses compared to control counterparts. Functional enrichments in this network were determined via STRING. n = 4/group*.

*GO, Gene Ontology; KEGG, Kyoto Encyclopedia of Genes and Genomes; PFAM, Protein Families*.

**Figure 6:**
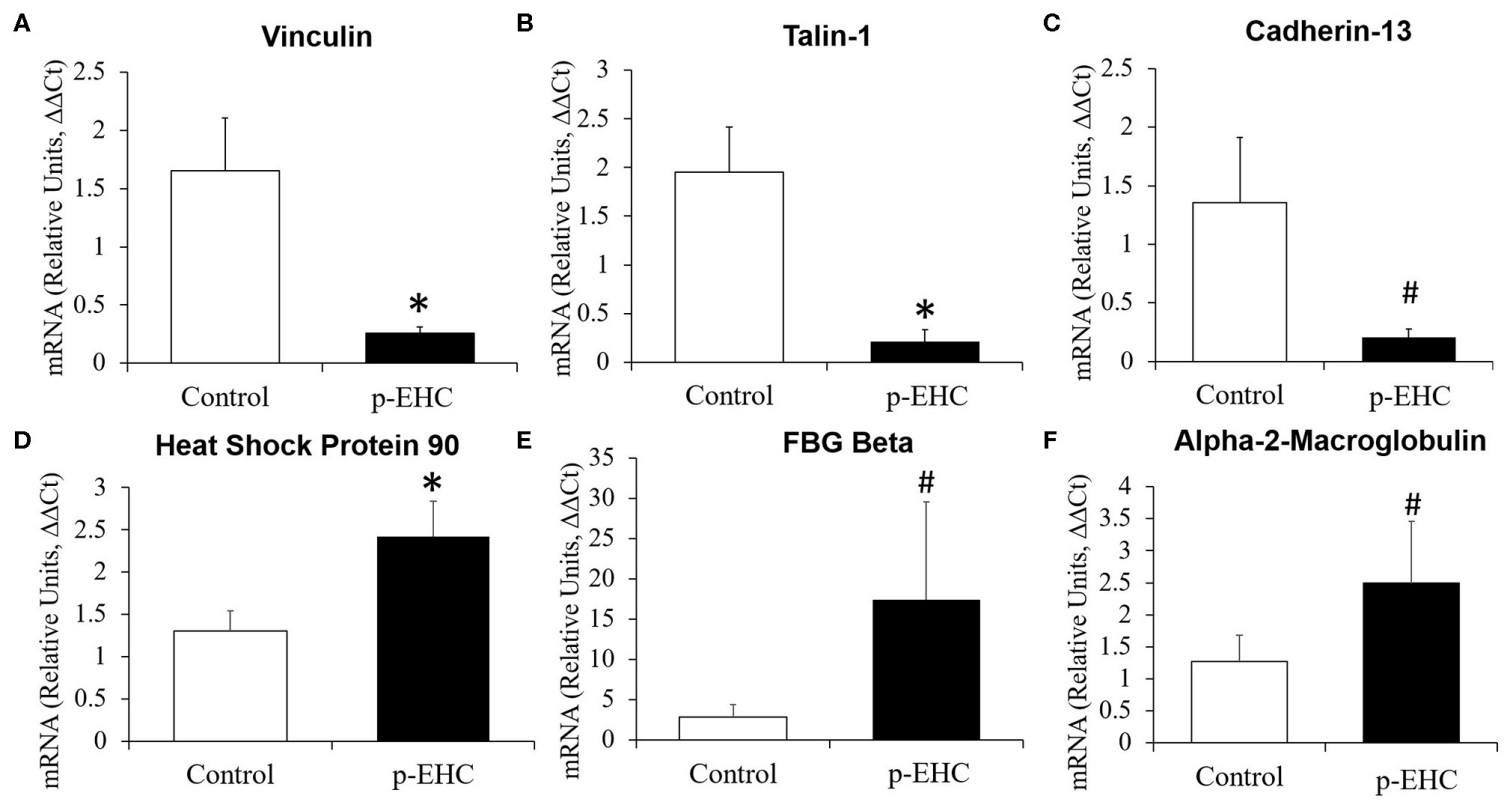
Mean fold change in messenger RNA (mRNA) expression of significantly differentially expressed proteins in lamellar tissue from healthy and hyperinsulinemic horses. mRNA expression of **(A)** vinculin, **(B)** talin-1, and **(C)** cadherin-13 were decreased in hyperinsulinemic (p-EHC) horses, while mRNA expression of **(D)** heat shock protein 90, **(E)** fibrinogen beta, and **(F)** alpha-2-macroglobulin were increased in hyperinsulinemic horses compared to controls. *n* = 4/group; *p < 0.05 vs. control, #*p* < 0.1 vs. control; statistical test: ΔΔCt method and two-tailed *t*-test.

Confirmatory experiments were performed by qRT-PCR of a subset of significantly differentially expressed proteins based on their potential importance to laminitis and metabolic diseases in other species (21–26). We determined that mRNA expression of vinculin, talin-1, and cadherin-13 were significantly downregulated in the treatment group as compared to the controls, and that heat shock protein 90, fibrinogen beta, and alpha-2-macroglobulin were significantly upregulated in the treatment group. Given the importance of these proteins in the cell-to-cell matrices and coagulation and complement response (27, 28), we assayed these gene's expression levels in control vs. laminitis samples via qRT-PCR. We observed that mRNA expression of the corresponding genes was also significantly altered in hyperinsulinemic horses. Using the ΔΔCT method and beta actin as the housekeeping gene, vinculin mRNA was downregulated by 84.5%, talin-1 mRNA was downregulated by 89.02%, and cadherin-13 mRNA was downregulated by 84.66% compared to controls. Similarly, mRNA expression of HSP90 was upregulated by 85.53% (*p* = 0.023), fibrinogen beta was upregulated by 510.4% (*p* = 0.11), and alpha-2-macroglobulin was upregulated by 97.32% (*p* = 0.14) (Tables 2, 3 and Figure 6).

## Discussion

Equine endocrinopathic laminitis is one of the most prevalent forms of laminitis, presenting a physically and economically debilitating disease which still presently has no cure (29, 30). The current study uses a shotgun proteomic analysis to explore potential mechanisms outside of the common steroid-, and inflammation-related pathways explored in the literature thus far. From this analysis, we have determined that in addition to the increase of inflammatory markers during insulin-induced laminitis, there is a decrease of significant cell-cell matrix and focal adhesion proteins as well as an increase of HSP90. These alterations in protein expression were confirmed by similar alterations in mRNA expression between groups.

In contrast with the lamellar tissue, we did not observe any difference in the cardiac proteomic profile of hyperinsulinemic horses compared to control horses. Using the same animal model of prolonged hyperinsulinemia-induced laminitis, we previously reported similar disparity between insulin-sensitive tissues and lamellar tissue (7). For instance, AKT-2, GSK-3β, GLUT1, and GLUT4 mRNA expression were upregulated in the hearts of hyperinsulinemic horses, suggesting that prolonged hyperinsulinemia induced an increase in insulin sensitivity in the heart, which could be cardioprotective. Conversely, as the primary source of disease pathogenesis during laminitis, we also here investigated the lamellar tissue. Consisting of lamellar interaction between dermis and epidermis of the hoof for structural integrity, this tissue has not been determined to be insulin-sensitive. While the heart is a very highly metabolically active organ and generally homogenous in constantly-contracting cardiomyocytes as the predominant cell type, the lamellae is a slow-growing mixture of proliferative epidermal cells, epidermal basal cells, and collagen-rich connective tissue. Since insulin resistance is not required for onset of insulin-induced laminitis (7), one could speculate that insulin-sensitive organs, such as the heart, are able to compensate for metabolic irregularities, especially in the short term, even while the lamellar tissue demonstrates a large increase in inflammation and damage to cell-to-cell structural integrity. Further studies using this unique equine model (15) could provide novel insights into cardioprotective mechanisms.

Endocrinopathic laminitis is often associated either with pituitary pars intermedia dysfunction, or, more commonly, hyperinsulinemia indicative of insulin resistance (29). Indeed, it has been previously demonstrated that, using the same animal model as in this study, a 48-h normoglycemic hyperinsulinemic protocol in an otherwise healthy horse is sufficient to produce Obel grade 2 laminitis in 100% of the animals receiving an hyperinsulinemic clamp (15). We have previously reported alterations of the expression of toll-like receptors and pro-inflammatory cytokines (31, 32), as well as alterations of glucose transport in striated muscle and adipose tissue of hyperinsulinemic horses (9, 33, 34). However, while we and others have described these significant mechanisms of tissue damage during this disease, identifying these mechanisms has not yet lead to substantial advances in treatment or prevention of endocrinopathic laminitis. Accordingly, we sought to identify new mechanisms or pathways which could be potential therapeutic targets and novel biomarkers. Current blood tests for proteins identified in this study are performed in horses and/or humans, including for fibrinogen, macroglobulins, and HSP90 (21, 24, 26). Thus, while our findings here in the lamellar tissue would need to be examined in the blood in relation to the detection of endocrinopathic laminitis, the potential for determining biomarkers, which could lead to early detection methods, is high.

While we identified a substantial number of proteins which were present in only either the control or hyperinsulinemic lamellar samples (but not both), the significantly differentially expressed proteins were present in both groups. In this study, we reported that protein expression of talin-1, vinculin, and cadherin-13, which are critically involved in cell-cell matrices and focal adhesions, are all significantly decreased in hyperinsulinemic horses compared to their control counterparts. Of note, these reductions may be occurring in both the lamellar tissue itself, as well as within the vascular support of the hoof, as these 3 proteins are significantly involved in vessel structure and homeostasis (22, 25). Syndecan-1, which is talin-dependent, is activated downstream of the insulin-like growth factor-1 receptor (IGF-1R), which is in turn activated by Syndecan-1 clustering (23). IGF-1R has been previously reported to be significantly higher in protein content of lamellar tissue vs. cardiac or skeletal muscle (7) and to be significantly decreased in insulin-treated horses compared to control counterparts (35). Given the significant reduction of talin-1 reported in the present study, this finding could be related to the previously reported alterations in lamellar IGF-1R during hyperinsulinemia. Additionally, to our knowledge, vinculin has not been reported as an underlying mechanism of hyperinsulinemia but is known to be a substrate of protein kinase C (36), which is part of the insulin signaling pathway. Again, the significant reduction of this protein could be intimately linked to the insulin dysregulation occurring in the lamellar tissue. Perhaps most importantly, cadherin-13 (also known as T-cadherin) has been demonstrated to be a regulatory component of insulin signaling endothelial cells, and may in fact be a determinant of the development of endothelial insulin resistance (37). Additionally, cadherin-13 may also modulate plasma levels of adiponectin in humans (38), low levels of which is closely linked to a major contributor to human insulin resistance (39) and which has similarly been reported to be significantly lower in horses and ponies prior to the development of clinical laminitis (40). Interestingly, cadherin-13 has been implicated in vascular disease and atherosclerosis (41) and others have found that cadherin-13 overexpression can promote insulin sensitivity while simultaneously reducing the ability to stimulate the Akt pathway (42). Finally, independent of its relationship with adiponectin, cadherin-13 appears to be necessary for the release of insulin both *in vitro* and *in vivo* (43). To our knowledge, the involvement of cadherin-13 or other focal adhesion proteins have not been previously implicated in the metabolic pathogenesis of endocrinopathic laminitis, and these may be important novel targets for future investigations.

We further here reported a significant increase of the protein expression of several important integrins, namely 3 fibrinogen isoforms (α, β, and γ), in the hyperinsulinemic group as compared to the control group. Recent studies reported a crosstalk between integrins and IGF-1R. For instance, some growth factor signaling cannot occur without specific integrin expression (44) and growth factors such as IGF-1R may act in response to specific action from integrins such as fibrinogen in a ligand-dependent manner (45). Additionally, IGF-1R inhibition has been reported to reduce fibrinogen binding in platelets of diabetic, but not healthy, mice (46). As fibrinogen is a significant participant in vascular homeostasis and tissue repair (47), vascular dysfunction has been reported to be associated with the underlying endocrinopathy of this disease (3). Although fibrinogen has been found to be upregulated in human diabetic patients (48), none of the fibrinogen isoforms have been significantly implicated in endocrinopathic laminitis without sepsis prior to this report (49).

Of note, alpha-2-macroglobulin may be a previously-unidentified participant in endocrinopathic laminitis disease pathogenesis. Alpha-2-macroglobulin is a known factor in coagulation, and thus its increase in the hyperinsulinemic hoof could be accounted for due to the tissue damage and inflammation during hyperinsulinemia-induced laminitis. Interestingly, alpha-2-macroglobulin has previously been reported to be increased in human diabetic patients (50). Similarly, hyperinsulinemia has been described as a hypercoagulable state, in part due to this increased thrombotic activity and the increased presence of alpha-2-macroglobulin (51). However, while the abundance of this protein was found to be increased in the plasma of horses with chronic laminitis (14), to our knowledge, this plasma protein has never before been described or implicated in equine metabolic syndrome. As alpha-2-macroglobulin is also capable of binding to growth factors, including insulin, this protein may be a potentially pathogenic factor during equine metabolic syndrome.

Finally, we found that heat shock protein 90 (HSP90) was significantly increased in the hyperinsulinemic horses compared to their control counterparts. While HSP90 has not been described in laminitic horses, the heat shock response has been found to be altered during type 2 diabetes in humans (52). For instance, HSP90 is higher in type 2 diabetic humans than in those with only impaired glucose tolerance (53), and inhibition of HSP90 appears to limit renal and vascular damage in diabetic mice (54). It is also worth noting that while laminitis is already well-associated with atherosclerotic markers, HSP90 inhibitors may reduce atherosclerosis during diabetes (55) and restore glucocorticoid sensitivity during Cushing's disease (56). Thus, the increased expression of HSP90 in the lamellar tissue of hyperinsulinemic horses with laminitis may be a significant detrimental contributor to the disease pathogenesis, and may also be a potential target for future therapeutic strategies. However, one could speculate that the alterations of the proteins discovered in this investigation could potentially be a result of disease pathogenesis, rather than a cause. Therefore, understanding these mechanistic relationships will require further investigation.

Significant alterations of major GO and KEGG pathways were identified by STRING analysis. Of note, we found a substantial increased expression of proteins involved in pathways involved in coagulation and complement cascades and platelet activation. An increase in the activity of coagulation and complement cascades, as well as platelet function, is in congruence with published studies regarding laminitis being characterizing by a highly inflammatory state (57). Similarly, the significant reduction of pathways involving focal adhesions is in accordance with the knowledge that laminitis is characterized by a degradation of the connective and structural tissues within the hoof (58). However, the increased expression of proteins involved in pathways related to ribosomal function, RNA binding, and extracellular exosomes, as well as the decreased expression of proteins involved with spliceosomes, are novel pathophysiological markers of this disease state. One could speculate that these alterations are predominately related to an increased inflammation and tissue damage, which will require further investigation.

In conclusion, using a proteomic analysis of equine cardiac and lamellar tissue, we reported a significant increase of alpha-2-macroglobulin, 3 fibrinogen isoforms and HSP90, as well as a significant reduction of several critical cell-cell matrix and focal adhesion participants, including talin-1, vinculin, and cadherin-13 in hyperinsulinemic compared to control horses. While many of these significantly differentially expressed proteins are described in other species during type 2 diabetes and metabolic syndrome, to our knowledge this is the first report in hyperinsulinemic horses with laminitis. While further insights in the regulation of these key proteins and their associated regulatory pathways is required, these proteins may represent novel therapeutic targets and biomarkers for hyperinsulinemic horses and the development of laminitis.

## Data Availability Statement

The datasets generated for this study can be found in the Digital Dryad, https://doi.org/10.5061/dryad.nk98sf7pz.

## Ethics Statement

The animal study was reviewed and approved by The Animal Ethics Committee of the University of Queensland (SVS/013/08/RIRDC).

## Author Contributions

AC, ML, SH, and VL: conceived and designed the experiments. AC and MWF: performed the experiments. AC, SH, and VL: analyzed the data. AC, SH, MOF, and VL: interpreted the data and edited the manuscript. AC and VL: wrote the manuscript. All authors approved of the final manuscript.

## Conflict of Interest

The authors declare that the research was conducted in the absence of any commercial or financial relationships that could be construed as a potential conflict of interest.
